# Early Mobilization after Cardiac Catheterization via Femoral Artery: A Systematic Review and Meta-Analysis

**DOI:** 10.31083/j.rcm2505152

**Published:** 2024-04-30

**Authors:** Jinyao Wang, Jun Cui, Shuangyan Tu, Qian Li, Ying Wang, Lihong Zhao, Zhonglan Chen, Yun Bao

**Affiliations:** ^1^Department of Cardiology, West China Hospital, Sichuan University/West China School of Nursing, Sichuan University, 610041 Chengdu, Sichuan, China; ^2^Department of Infrastructure, West China Hospital, Sichuan University, 610041 Chengdu, Sichuan, China; ^3^Department of Neurology, West China Hospital, Sichuan University/West China School of Nursing, Sichuan University, 610041 Chengdu, Sichuan, China; ^4^Center of Gerontology and Geriatrics, West China Hospital, Sichuan University/West China School of Nursing, Sichuan University, 610041 Chengdu, Sichuan, China; ^5^Department of Radiology, West China Hospital, Sichuan University/West China School of Nursing, Sichuan University, 610041 Chengdu, Sichuan, China

**Keywords:** early mobilization, trans-femoral cardiac catheterization, ERAS, systematic review

## Abstract

**Background:**

Early mobilization is one of the essential components of enhanced recovery after surgery (ERAS) pathways and has been shown to reduce complications and optimize patient outcomes. However, the effect of early mobilization for patients who undergo trans-femoral cardiac catheterization and the time for optimal mobilization timing remains controversial. We aimed to identify the safety of early mobilization and provide the optimum timing for early mobilization for patients undergoing trans-femoral cardiac catheterization.

**Methods:**

We searched MEDLINE, EMBASE, PubMed, Web of Science, Cochrane databases of systematic reviews, CINAHL, SCOPUS, China National Knowledge Infrastructure (CNKI), Wan Fang Database, and Chinese Science and Technology Periodical Database (VIP) comprehensively for randomized controlled trials associated with early mobilization, to explore its effects on patients after a trans-femoral cardiac catheterization. The risk of bias and heterogeneity of studies was assessed using the Revised Cochrane risk-of-bias tool for randomized trials (RoB 2) and $I^{2}$ index, respectively. The comprehensive Meta-analysis (CMA) was adopted to perform the meta-analysis.

**Results:**

We identified 14 trials with 2653 participants. Early mobilization was associated with significant decrease in back pain (mean difference (MD) = 0.634, 95% CI: 0.23–1.038; *p* = 0.002), especially in patients receiving instruction for early mobilization in 3 h~4 h versus 5 h~6 h (MD = 0.737, 95% CI: 0.431–1.043; *p* = 0.000) and 12 h versus 24 h (OR = 5.504, 95% CI: 1.646–18.407; *p* = 0.006) categories. The results of subgroup analysis also showed a significant risk reduction in urinary retention by early mobilization in 12 h versus 24 h (OR = 5.707, 95% CI: 1.859–17.521; *p* = 0.002) category.

**Conclusions:**

Early mobilization has not been shown to increase the risk of bleeding, hematoma, pseudoaneurysm, urinary retention, and pain at the puncture site after trans-femoral cardiac catheterization. Early mobilization is a practical initiative in ERAS, and it may be safe and feasible to advance the mobilization to 2 h~4 h.

## 1. Introduction

Cardiac catheterization, a minimally invasive procedure accompanied by cardiac catheters placed into vessels, has progressed to encompass a wide range of heart diagnostic and therapeutic procedures, including hemodynamic assessment, coronary and peripheral arterial angiography and intervention, and structural heart disease intervention [1, 2]. Currently, diagnostic and therapeutic heart catheterizations are common for electively or emergent procedures for patients with cardiovascular symptoms [3, 4, 5].

Femoral access remains a preferred vascular access site for cardiac catheterization with less radiation and contrast than trans-radial access, especially for complex coronary interventions. It is also the predominant approach for transcatheter aortic valve replacement [6, 7, 8]. In these procedures, manual or mechanical application of a firm pressure above the puncture site and restricted bed rest in a supine position with the affected leg immobilization after sheath removal are essential [9, 10]. Bed rest after trans-femoral cardiac catheterization is necessary to promote the healing of the puncture site and prevent minor to severe complications, including arterial bleeding, hematoma, pseudoaneurysms, and other vascular complications [11, 12]. Nevertheless, long-term bed rest is associated with numerous cardiovascular, pulmonary, and muscular complications [13, 14, 15, 16, 17]. Many patients who lie in bed without changing position for a long time complain of back pain or urinary discomfort, which can result in increased medical costs due to prolonged hospital stay [18, 19].

Shortening the length of bed rest after trans-femoral catheterization may result in improved outcomes following cardiac catheterization procedures. Early mobilization, one of the countermeasures to decrease bed rest complications, has been proven to be a feasible and safe intervention to reduce hospital stay, venous thrombosis and embolisms, and falls [20, 21, 22]. However, there is controversy regarding the evidence of optimal time for mobilization following trans-femoral cardiac catheterization. The duration of bed rest after sheath removal ranges from 1 h to 24 h according to the different catheter sizes, the dose of heparin used, and the techniques and protocols in various cardiac centers [23, 24, 25]. Chair *et al*. [26] indicated that the length of bed rest for trans-femoral cardiac catheterization could decrease from 12–24 h to 4 h. Gall *et al*. [27] demonstrated that a bed rest duration of 1.5 h in restricted posting was not associated with increased complications. Several studies using arterial closure devices for femoral artery puncture sites also confirmed the feasibility of 6–8 h to ambulation [28, 29]. A recent network meta-analysis showed that ambulation could be safely implemented as early as 2 hours after trans-femoral catheterization [30]. Unfortunately, no existing studies involved Chinese patients, and its applicability to Chinese patients remains unclear. This systematic review aimed to summarize recommendations regarding the optimum timing for early mobilization and to identify the safety of early mobilization for patients who underwent trans-femoral cardiac catheterization.

## 2. Methods

### 2.1 Study Selection and Search Strategy

We searched the MEDLINE, EMBASE, PubMed, Web of Science, Cochrane databases of systematic reviews, CINAHL, SCOPUS, China National Knowledge Infrastructure (CNKI), Wan Fang Database, and Chinese Science and Technology Periodical Database (VIP) for all relevant studies from the earliest data available to December 2022. The search and reporting procedure followed the Preferred Reporting Items for Systematic Reviews and Meta-Analyses (PRISMA) 2020 checklist [31]. In addition, we manually retrieved and evaluated the reference lists of all the identified studies. All contents and methods were approved by the ethics committee of West China Hospital, Sichuan University (2021, Review No. 591).

Two independent researchers (JYW and JC) performed all searches. Our search strategy was based on the medical subject headings (Mesh) and free-text words, and the main Mesh were as follows: ‘Cardiac Catheters’, ‘Femoral Artery’, ‘early mobilization’, ‘Walking’ and ‘Bed Rest’. Two reviewers (JYW and SYT) independently screened and identified the studies for potential eligibility. We consulted the corresponding author to get a consensus about any controversy. The complete search strategy is shown in **Supplementary Material**.

### 2.2 Study Inclusion and Exclusion Criteria

The PICOS framework (P for the population of interest, I for intervention, C for comparison group, O for outcome, and S for study design) guided the study process [32, 33].

Studies that met the following inclusion criteria were included in this meta-analysis: (1) heart catheterization via femoral artery approach, (2) human studies comparing the safety of different lengths of bed rest with or without altering the patient’s position or the angle of beds, (3) participants older than 18 years of age, (4) randomized or quasi-randomized controlled trials involving more than ten patients in each group, (5) the studies provided no less than one clinical outcome, including bleeding, hematoma, pseudoaneurysm at the puncture site, back pain, urinary retention, or bladder catheterization, (6) language: English and Chinese. There were no restrictions concerning patient characteristics or healthcare settings.

We excluded studies if (1) any vascular closure device (VCD) or coagulants was applied to achieve the puncture site hemostasis except a bandage, sandbag, or manual compression, (2) the full text was not available, (3) only the position of the patients or the angle of the beds was altered, but the patients did not get out of bed, (4) the effects to combine early mobilization with other intervention variables (e.g., encouraging exercise and applying ice packs), (5) there was no specific ambulation timing or measurable outcome.

### 2.3 Type of Intervention and Outcomes

Shortening the post-cardiac catheterization duration of bed rest was the early mobilization group, and the longer duration of bed rest was regarded as the late ambulation group. According to the characteristics of the included studies, we classified different bed rest duration into the following four comparison subgroups: Group A: Comparing 2 h versus 4 h~6 h of bed rest; Group B: Comparing 3 h~4 h versus 5 h~6 h of bed rest; Group C: Comparing 4 h~6 h versus greater than or equal to 8 h of bed rest; Group D: Comparing 12 h versus 24 h of bed rest. The primary outcomes of interest were the incidence of bleeding and hematoma at the puncture site. The secondary outcomes were the incidence of back pain, pseudoaneurysm, urinary retention, and pain at the puncture site.

### 2.4 Data Extraction

Using Excel, two authors (JYW and SYT) independently extracted and coded data from the qualified studies into standard tables. The original authors would be contacted for further information if data from the included studies were insufficient. Any disagreement was verified by the corresponding author. The items included: authors, year of publication, country, setting and location of the study, number of patients, the mean age of patients, study design, duration of the bed rest, hemostasis method, outcomes, and other relevant information.

### 2.5 Quality and Risk of Bias Assessment

The risk of bias in the included studies was independently evaluated by two authors (JYW and SYT) using the RoB 2 tool [34]. This tool assesses the following key areas of potential bias: randomization methods; deviations from intended intervention; missing outcome data; measurement of outcome; and selection of reported results. The judgment for the domain of RoB 2 is generated by an algorithm and can be ranked as low, high, or show some concerns. Each study is also given an overall judgment of RoB 2 based on the same options [34]. The corresponding author was available for arbitration in any disagreement regarding the ranking process and results.

### 2.6 Data Synthesis and Statistical Analysis

This study was statistically analyzed using the Comprehensive Meta-Analysis software, Version 2 (Biostat, Englewood, NJ, USA). We pooled the odd ratio (OR), mean difference (MD), and 95% confidence intervals (CI) from separate studies to assess the intended effect sizes. Cochran’s Q test and the degree of inconsistency ($I^{2}$ index) were used to evaluate the heterogeneity across studies. There was no heterogeneity if the $I^{2}$ statistic was less than 25%, a low heterogeneity if the $I^{2}$ statistic was 25–50%, and a moderate heterogeneity if the $I^{2}$ statistic was 50–75%, while the $I^{2}$ > 75% reflected a high heterogeneity [35]. Funnel plots, Egger’s test [36], and Begg’s test [37] were used to analyze publication bias. Random-effect models that reflected the differences between each study were applied because of the heterogeneity across studies [38]. Subgroup analyses were performed based on the different bed rest duration. A *p*-value < 0.05 was considered statistically significant.

## 3. Result

### 3.1 Selection of Studies

**Fig. 1.**
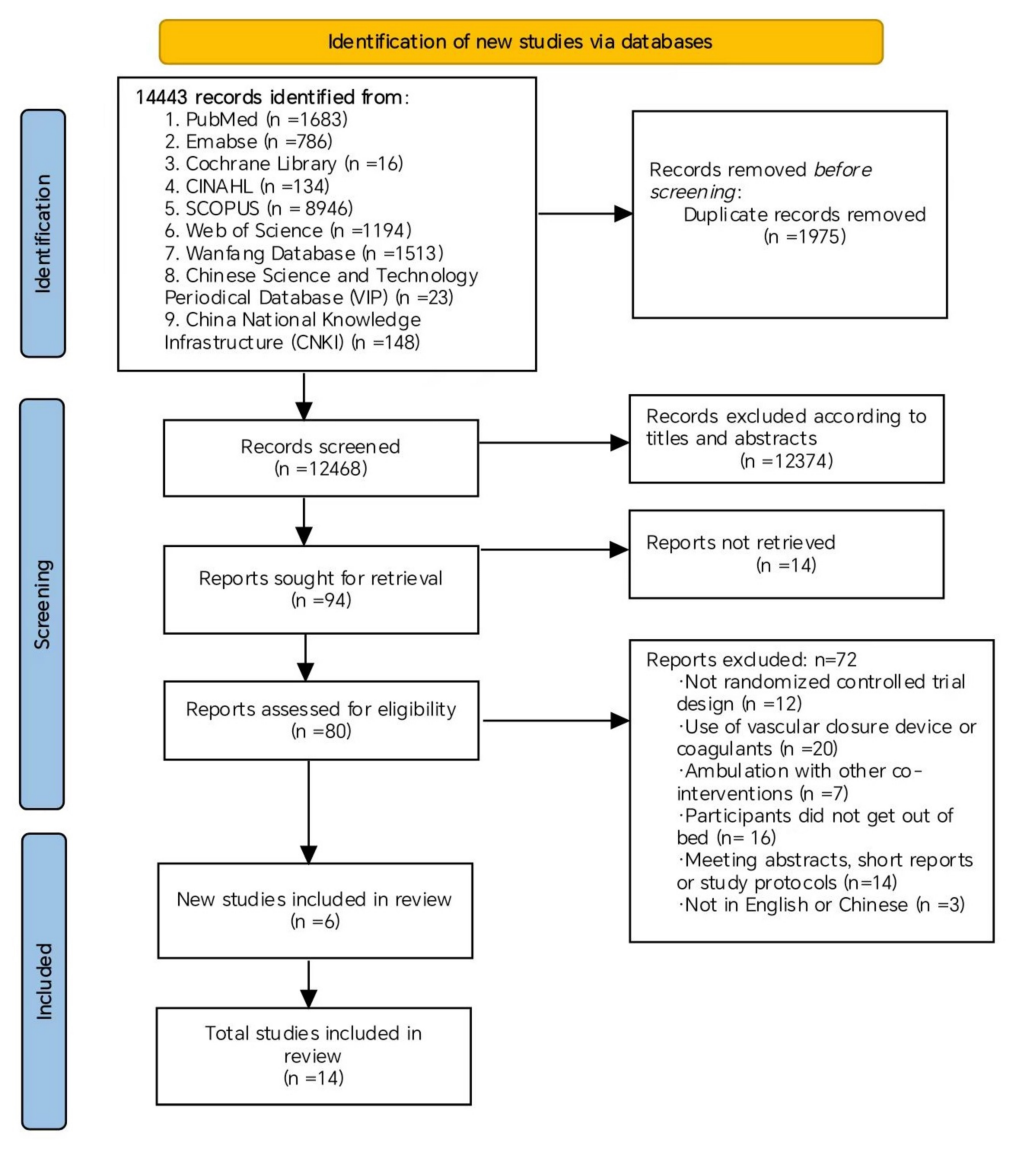
**Flow diagram of the systematic searching process**.

The initial literature search yielded 14,443 studies, of which 1975 studies were excluded after removing duplicates. There were 94 studies eligible for further evaluation after 12,374 studies were excluded because of irrelevant titles and abstracts. At the full-text screening stage, only 80 studies were reviewed because 14 studies did not have the full text, and six studies met the inclusion and exclusion criteria. Then, we added eight eligible studies from the reference review process. Finally, 14 studies with 2653 participants were selected for this meta-analysis. A summary of the PRISMA flow diagram of the study selection process is shown in Fig. 1.

### 3.2 Selected Studies and Characteristics

The 14 randomized controlled trials involving 2653 participants included 2 Chinese and 12 English studies. Two [39, 40] of the fourteen included studies were assigned to Group A with 325 participants, five studies [23, 41, 42, 43, 44] belonged to Group B with 1388 participants, five [26, 29, 45, 46, 47] were allocated to Group C with 705 participants, and the remaining two Chinese studies [48, 49] were in Group D with 235 participants. The mean age of the participants was between 53–67 years. These researches took place across three continents, five studies in North America, seven in Asia, and two in South America, of which four were conducted in China and four in the USA. All studies except for one conducted by Gu *et al*. [48] reported two arms. Based on our subgroup rules, we only selectively extracted the data from two groups (ambulation after 12 h versus 24 h). Six studies [23, 29, 42, 45, 46, 48] reported the indication for the cardiac catheterization, three for diagnosis [23, 42, 45] and three for therapy [29, 46, 48], respectively. Nine studies [23, 29, 40, 41, 42, 43, 46, 47, 48] used the manual compression method to obtain hemostasis, and a few studies employed additional methods such as bandage dressing or sandbags. Regarding heparin and sheath size, the usage regimen varied among the studies with some unavailable data. The characteristics are shown in Table 1 (Ref. [23, 26, 29, 39, 40, 41, 42, 43, 44, 45, 46, 47, 48, 49]).

**Table 1.** **Summarized characteristics of the included studies**.

Author (year)	Participants	Randomization method	Mean age		Time to ambulation		Catheterization	Heparin usage in the procedure	Hemostasis method	Outcomes
Country			IG	CG	IG	CG	Type and sheath size			
Augustin *et al*. (2010) [41]	TN = 347	Random computer-generated list	59.7 ± 9.9	61 ± 10.4	3 h postprocedure	6 h postprocedure	Elective PCI	Intravenous heparin in the dose of 100 UI/kg	Manual compression	①②④⑤⑦⑧
Brazil	CG: N = 175						6 F			
	IG: N = 172									
Baum *et al*. (1996) [39]	TN = 205	NA	58 ± 10	59 ± 10	2 h postprocedure	4 h postprocedure	Cardiac catheterization	IG: 94 UI	NA	①②
USA	CG: N = 104						5 F–8 F	CG: 96 UI		
	IG: N = 101									
Chair *et al*. [26] (2007)	TN = 86	Computer-generated random table of number	62.7 ± 9.7	63.2 ± 9.7	4 h postprocedure	12–24 h postprocedure	Elective cardiac catheterization	NA	NA	①②③⑨
Hong Kong China	CG: N = 43						NA			
	IG: N = 43									
Chair *et al*. (2012) [45]	TN = 137	Computer-generated random list	NA	NA	4 h postprocedure	12–24 h postprocedure	Elective diagnostic cardiac catheterization	NA	NA	①②③⑨⑩⑪
Hong Kong China	CG: N = 74						NA			
	IG: N = 63									
Farmanbar *et al*. (2008) [40]	TN = 120	NA	60.17 ± 11.5	59.9 ± 10.15	2 h postprocedure	6 h postprocedure	Angiography	NA	Manual compression + transparent dressing + sandbag	①②④⑫
Iran	CG: N = 60						7 F			
	IG: N = 60									
Fowlow *et al*. (1995) [29]	TN = 85	NA	MA of males: 54; MA of females: 61	MA of males: 58.2; MA of females: 63.9	6 h postprocedure	8 h postprocedure	Elective PTCA	Average usage was 10,125 UI	Manual compression + pressure dressing	①②⑫⑬
Canada	CG: N = 44						7.5 F–9 F			
	IG: N = 41									
Bogart *et al*. (1999) [23]	TN = 200	NA	60 ± 10	55 ± 10	4 h postprocedure	6 h postprocedure	Diagnostic cardiac catheterization	NA	Manual compression	①②④⑫⑭⑮
USA	CG: N = 100						8 F			
	IG: N = 100									
Matte *et al*. (2016) [42]	TN = 730	Computer-generated random list	61.5 ± 11	63 ± 10	3 h postprocedure	5 h postprocedure	Diagnostic cardiac catheterization	NA	Manual compression	①②④⑥⑦⑳
Brazil	CG: N = 363						6 F			
	IG: N = 367									
Moeini *et al*. (2010) [46]	TN = 124	Admission numbers	NA	NA	4 h postprocedure	8 h postprocedure	Angioplasty	72–100 UI/kg	Manual compression + sandbag	①②
Iran	CG: N = 62						7 F			
	IG: N = 62									
Pooler-Lunse *et al*. (1996) [43]	TN = 29	NA	60	64.6	4 h postprocedure	6 h postprocedure	Cardiac angiography	Heparin doses averaged between 900–1200 UI per hour	Manual compression + pressure dressing	①②③
Canada	CG: N = 15						6 F–8 F			
	IG: N = 14									
Wang *et al*. (2001) [44]	TN = 82	NA	58.7	62	4 h postprocedure	6 h postprocedure	Cardiac catheterization	NA	Sandbag + adhesive bandage	①②③⑥⑪⑯
USA	CG: N = 41						5 F/6 F			
	IG: N = 41									
Gu *et al*. (2015) [48]	TN = 145	NA	IG1: 67 ± 10.5; IG2: 64.8 ± 11	65.2 ± 9.8	IG1: 18 h postprocedure	24 h postprocedure	Therapeutic cardiac Catheterization	CG: 7177 ± 4234 UI	Manual compression + pressure dressing + sandbag	①③⑤⑦⑰
China	CG: N = 48				IG2: 12 h postprocedure		6 F	IG1: 6306 ± 4432 UI		
	IG1: N = 49							IG2: 6865 ± 4544 UI		
	IG2: N = 48									
Yuan (2013) [49]	TN = 90	NA	NA	NA	12 h postprocedure	24 h postprocedure	Cardiac catheterization	NA	Adhesive bandage + sandbag	①②③⑤⑱
China	CG: N = 45									
	IG: N = 45									
Lau *et al*. (1993) [47]	TN = 273	National identity card numbers	53 ± 11	55 ± 11	6 h postprocedure	The following morning postprocedure	Cardiac catheterization	2000–2500 UI	Manual compression	②⑲
Singapore	CG: N = 131						7 F			
	IG: N = 142									

Notes: TN, total number; CG, control group; IG, intervention group; NA, not available; MA, mean age; ①, bleeding; ②, Hematoma; ③, back pain; ④, pseudoaneurysm; ⑤, urinary retention; ⑥, puncture-site pain; ⑦, vasovagal response; ⑧, lumbar pain; ⑨, urinary discomfort; ⑩, general well-being; ⑪, patient satisfaction; ⑫, arteriovenous fistula; ⑬, Pain perception; ⑭, limb ischemia; ⑮, thrombosis of the femoral artery; ⑯, numbness or tingling in affected leg; ⑰, anxiety; ⑱, insomnia; ⑲, allergy; ⑳, bruising; PCI, percutaneous coronary intervention; PTCA, percutaneous Transluminal Coronary Angioplasty; UI, units.

### 3.3 Critical Appraisal of the Included Studies

**Fig. 2.**
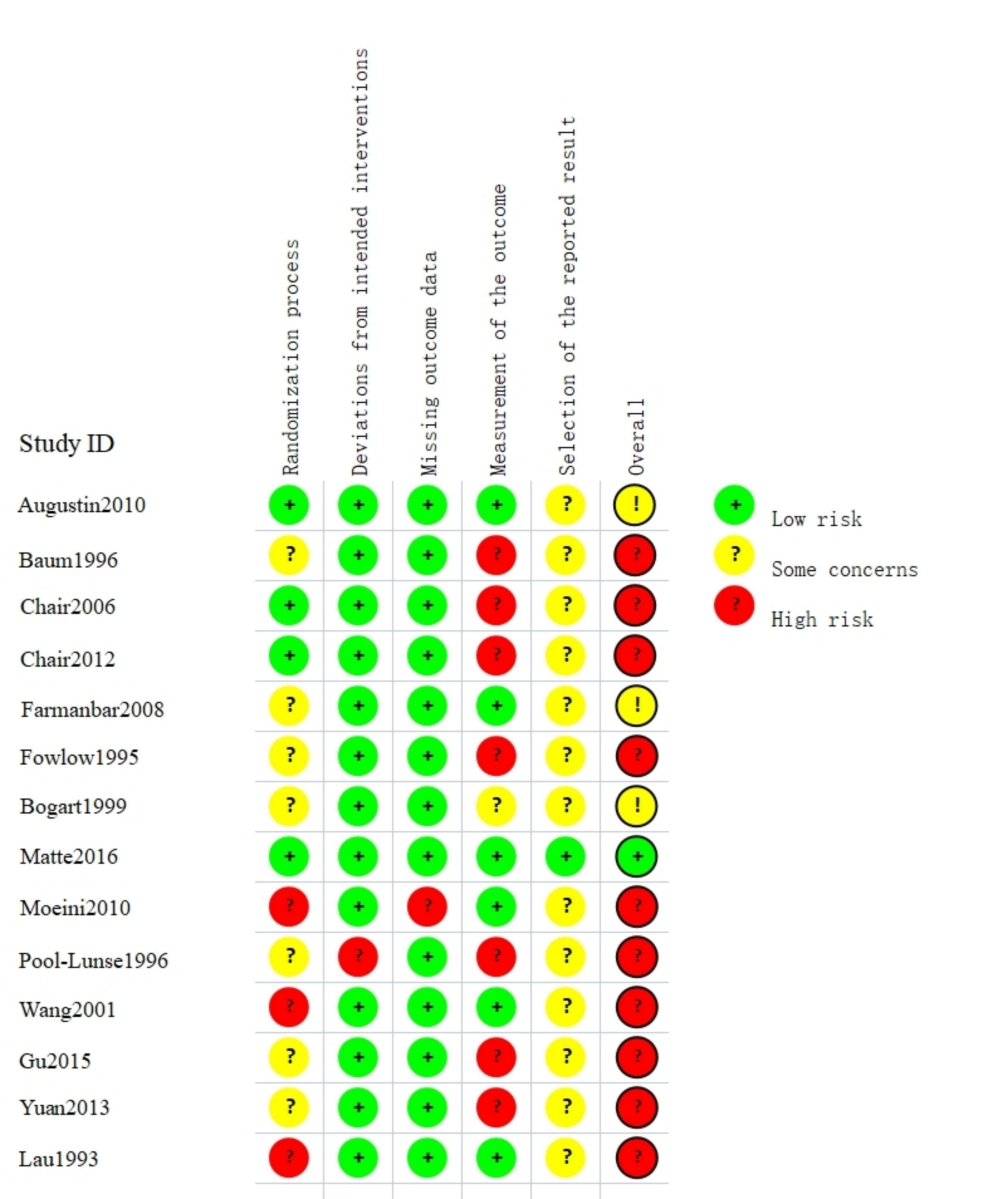
**Risk of bias (RoB 2) assessment plot for the included randomized controlled trial studies**.

**Fig. 3.**
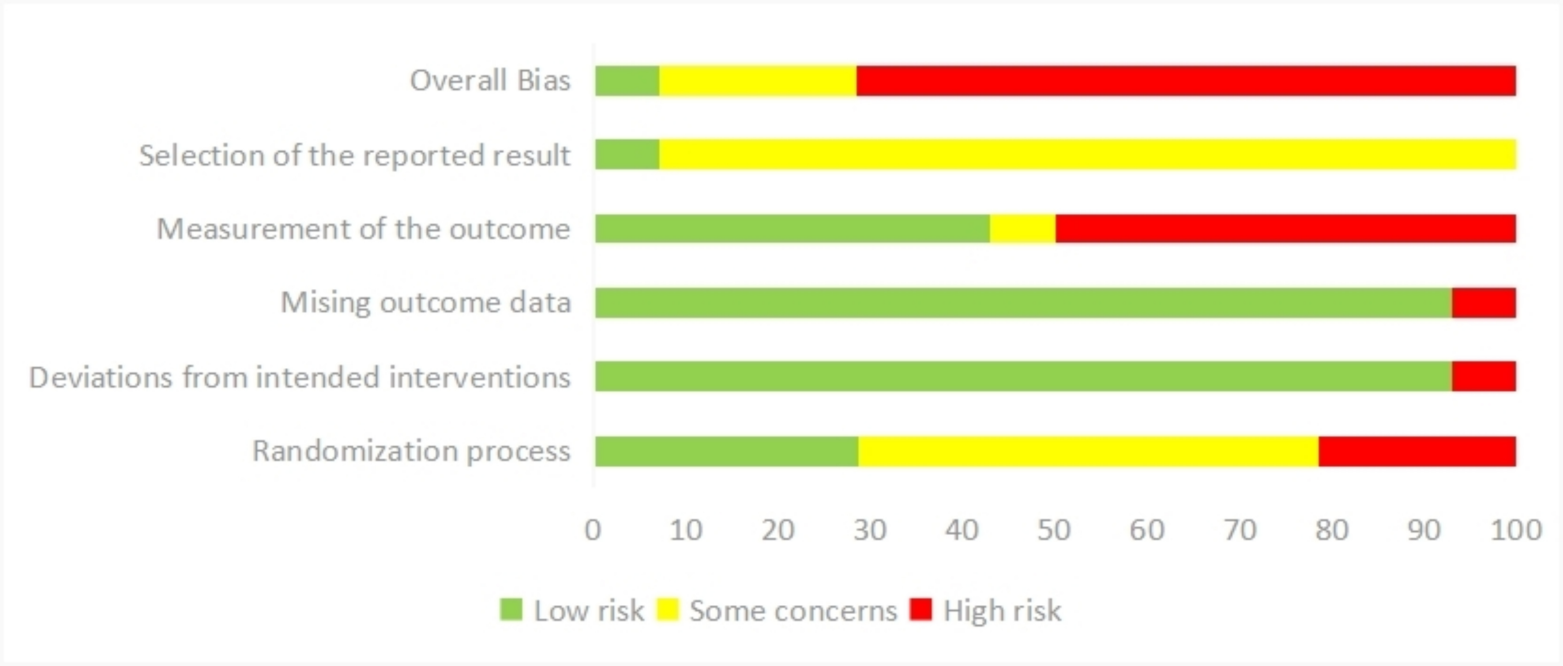
**Weighted summary plot of all the types of bias from the included studies**.

Two authors (JYW and SYT) independently judged the risk of bias. Overall, ten of the fourteen studies were ranked as “high risk” and only one randomized controlled trial (RCT) was rated as “low risk”. Matte *et al*. [42] reported the research design and process according to all the domains of RoB 2, so we regarded this study as “low risk”. Three RCTs were judged as having some concerns considering overall risk [23, 40, 41]. Patients were doomed to be instructed about resting duration due to the nature of the study design; therefore, allocation concealment could not be achieved. We did not perceive any studies as having a high blinding risk of bias for participants or the individuals delivering the interventions. All the studies reported that patients followed the bed rest instructions. But in the deviations from intended interventions domain, only one study was scored as “high risk” due to not conforming to the established hemostatic protocol [43]. In the missing outcome data domain, we rated one study by Moeini *et al*. [46] to be at “high risk” because of the vague reporting of the study results. Figs. 2,3 depict the assessment results.

### 3.4 The Effects of Early Mobilization on the Different Outcomes

**Fig. 4.**
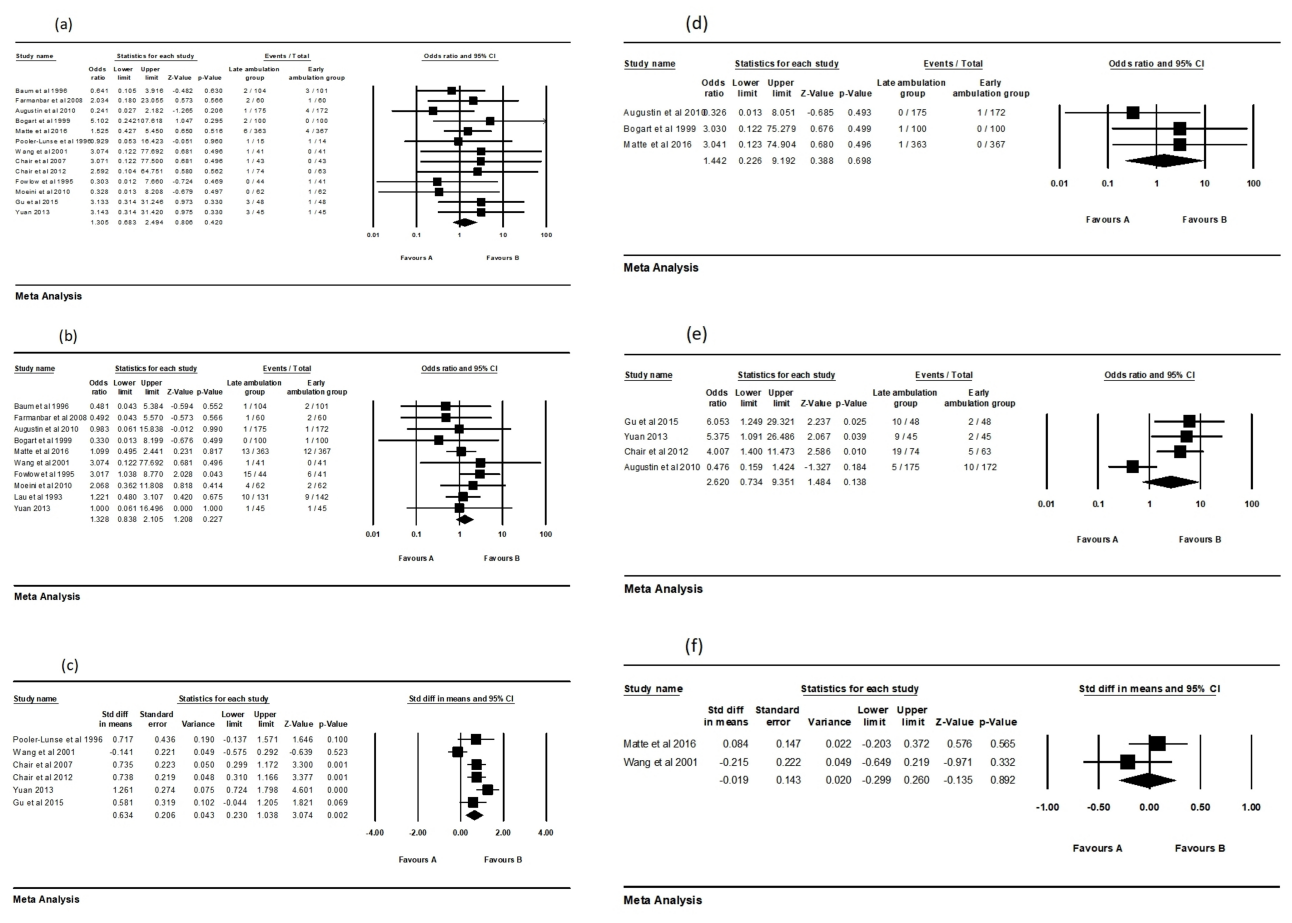
**The results of the overall odds ratio or mean difference for early mobilization effect among different outcomes**. (a) Bleeding. (b) Hematoma. (c) Back pain. (d) Pseudoaneurysm. (e) Urinary retention. (f) Pain at the puncture site. The horizontal lines denote the 95% CI, the Square (◼) shows the point estimate (the size of the square corresponds to its weight); the diamond shows (◆) the combined overall effects of ambulation at 95% CI. CI, confidence interval.

We adopted a random effects analysis to assess the effect sizes. The forest plot showed the pooled effects of the six outcomes (bleeding, hematoma, back pain, pseudoaneurysm, urinary retention, and pain at the puncture site). In summary, six RCTs with a sample size of 569 found that the back pain of patients was significantly reduced by early mobilization (MD = 0.634, 95% CI: 0.23–1.038; *p* = 0.002). Nevertheless, early mobilization did not have any significant effects on bleeding (OR = 1.305, 95% CI: 0.683–2.494; *p* = 0.42), hematoma (OR = 1.328, 95% CI: 0.838–2.105; *p* = 0.227), pseudoaneurysm (OR = 1.442, 95% CI: 0.226–9.192; *p* = 0.698), urinary retention (OR = 2.62, 95% CI: 0.734–9.351; *p* = 0.138), and pain at the puncture site (MD = –0.019, 95% CI: –0.299–0.26; *p* = 0.892) among patients who underwent cardiac catheterization. The overall results of the meta-analysis are shown in Fig. 4.

### 3.5 Subgroup Analysis

Subgroup analyses were not possible owing to the lack of different bed rest duration groups concerning the outcome of pseudoaneurysm and pain at the puncture site. We only conducted subgroup analyses based on four outcomes: bleeding, hematoma, back pain, and urinary retention. The moderator variable was bed rest duration, including group A (2 h versus 4 h~6 h), group B (3 h~4 h versus 5 h~6 h), group C (4 h~6 h versus ≥8 h), and group D (12 h versus 24 h). Some subgroup analyses were unavailable because of the limited studies regarding different bed rest duration for each outcome.

**Fig. 5.**
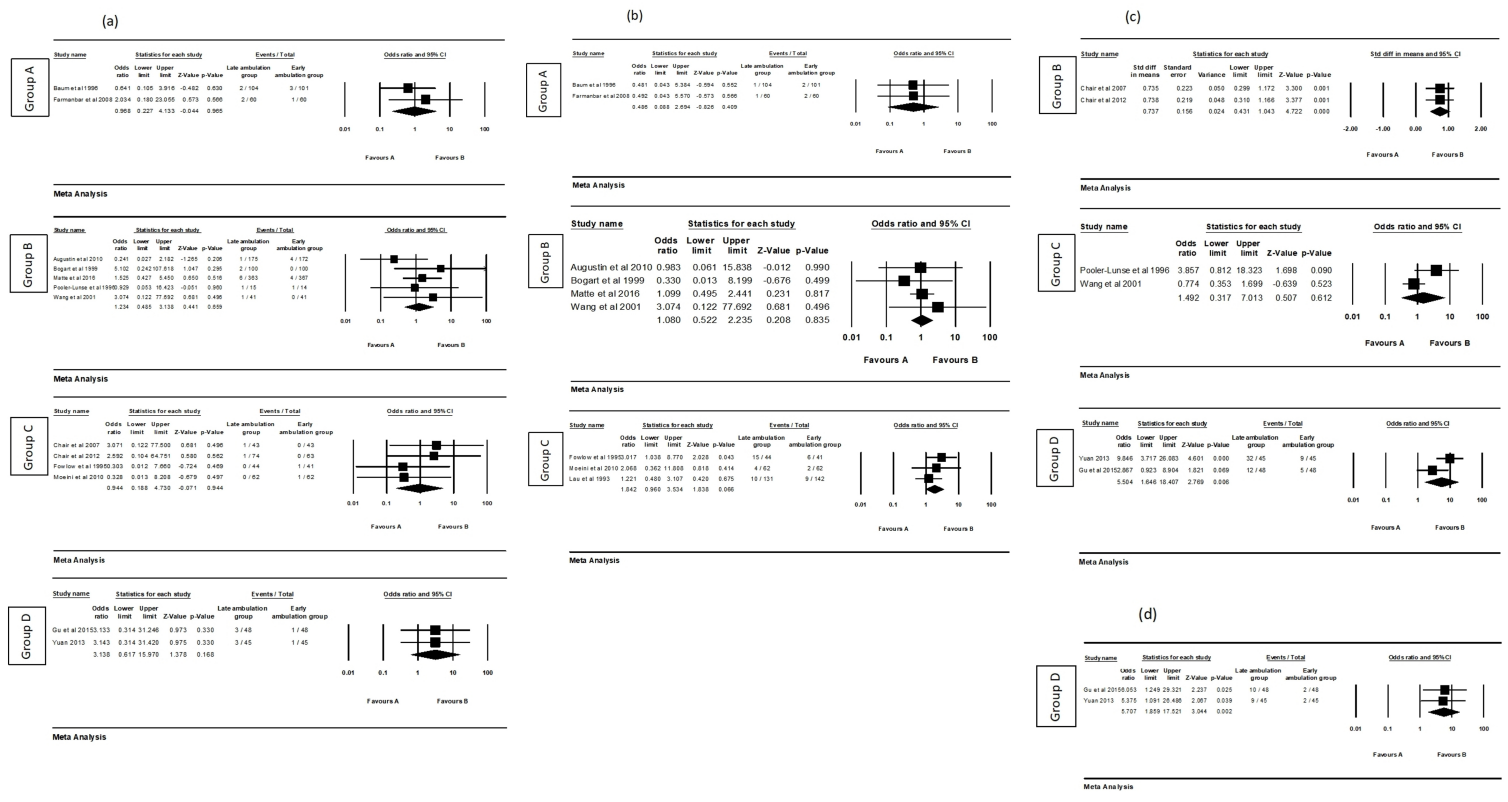
**Subgroup analysis to evaluate the effect of early mobilization**. (a) Bleeding. (b) Hematoma. (c) Back pain. (d) Urinary retention. Group A, 2 h versus 4 h~6 h; Group B, 3 h~4 h versus 5 h~6 h; Group C, 4 h~6 h versus ≥8 h; Group D, 12 h versus 24 h. The horizontal lines denote the 95% CI, the Square (◼) shows the point estimate (the size of the square corresponds to its weight); the diamond shows (◆) the combined overall effects of ambulation at 95% CI. CI, confidence interval.

We found the effect of patients’ back pain were statistically significant when they were they had instruction for early mobilization in groups B (MD = 0.737, 95% CI: 0.431–1.043; *p* = 0.000) and D (OR = 5.504, 95% CI: 1.646–18.407; *p* = 0.006). The forest plot shows the patients’ urinary retention was significantly decreased by early mobilization in group D (OR = 5.707, 95% CI: 1.859–17.521; *p* = 0.002). In contrast, early mobilization in group C (OR = 1.492, 95% CI: 0.317–7.013; *p* = 0.612) did not have an effect on the patients’ back pain. As for outcomes of bleeding and hematoma, the results showed no statistical significance among all the bed rest duration groups. Fig. 5 shows the details of the subgroup analysis.

### 3.6 Sensitivity and Publication Bias

**Fig. 6.**
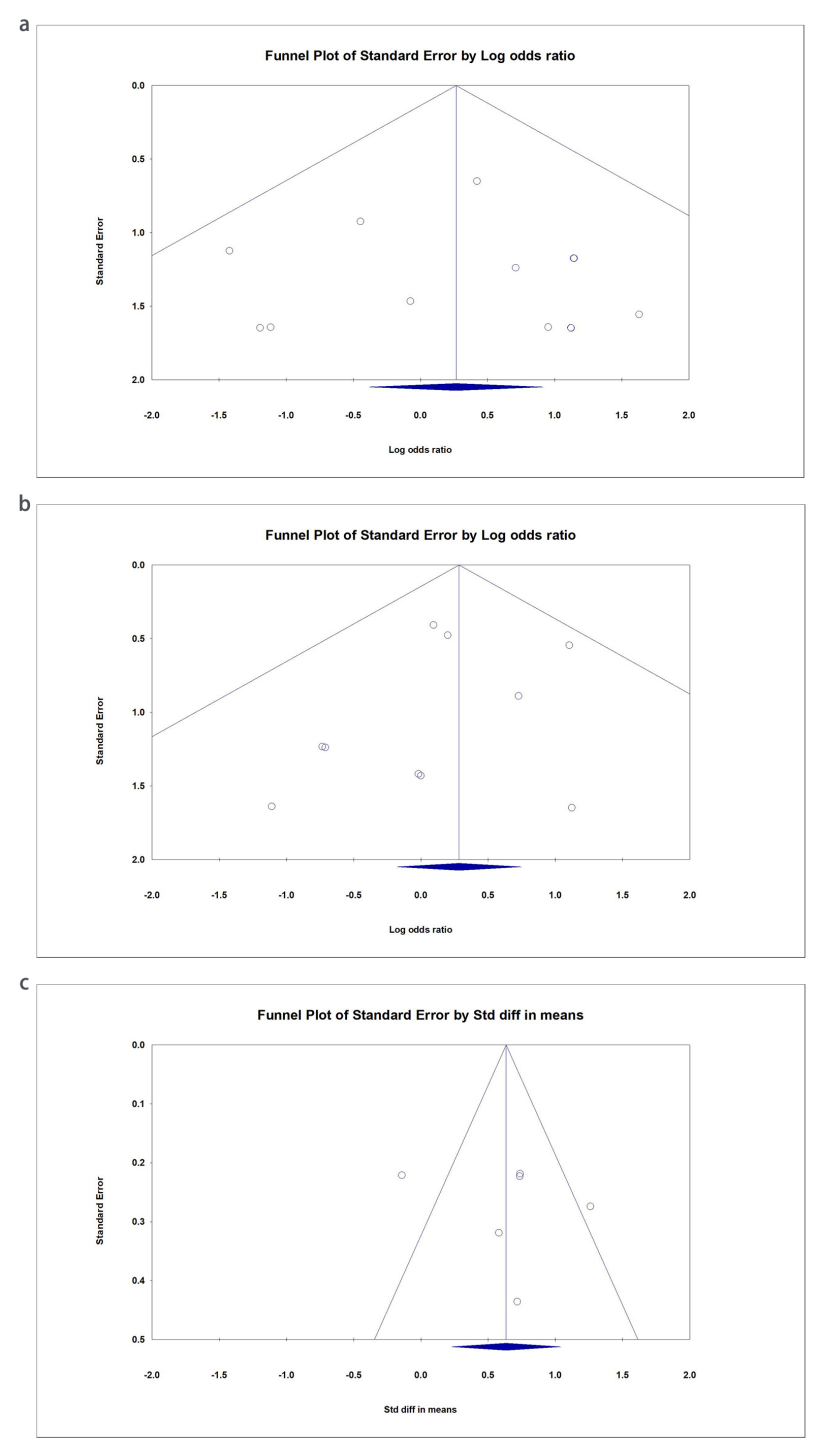
**Funnel plot to assess publication bias in the effects of early ambulation on different outcomes**. (a) Bleeding. (b) Hematoma. (c) Back pain. Diagonal lines represent pseudo-95% confidence intervals. The y-axis represents the standard error (weight in the pooled analysis), the x-axis indicates the effect size, and the vertical line shows the calculated estimated effect of different outcomes.

The $I^{2}$ index of the effects of early mobilization on bleeding ($I^{2}$ = 0%), hematoma ($I^{2}$ = 0%), pseudoaneurysm ($I^{2}$ = 0%), and pain at the puncture site ($I^{2}$ = 21.31%) did not reflect heterogeneity, whereas the results of back pain ($I^{2}$ = 72.06%) and urinary retention ($I^{2}$ = 73.99%) showed a moderate heterogeneity. The publication bias of included studies reporting bleeding, hematoma, and back pain could be estimated by funnel plots, as shown in Fig. 6. Random-effect models were used in the overall meta-analysis process due to insufficient studies regarding some outcomes. The *p*-value of Egger’s test and Begg’s test for the effect of bleeding (*p*-value = 0.831 for Egger’s test and *p*-value = 0.714 for Begg’s test), hematoma (*p*-value = 0.415 for Egger’s test and *p*-value = 0.788 for Begg’s test), back pain (*p*-value = 0.621 for Egger’s test and *p*-value = 0.851 for Begg’s test), pseudoaneurysm (*p*-value = 0.941 for Egger’s test and *p*-value = 0.602 for Begg’s test), and urinary retention (*p*-value = 0.476 for Egger’s test and *p*-value = 0.497 for Begg’s test) did not indicate significant publication bias. Still, the possibility of publication bias cannot be denied. As shown in Table 2.

**Table 2.** **The summary of publication bias results**.

Outcomes	Bed rest duration group	Number of studies	Sample size	$I^{2}$ (%)	Egger’s test	Begg’s test
Bleeding	/	13	2380	0	0.831	0.714
	2 h versus 4 h~6 h	2	325	0	/	/
	3 h~4 h versus 5 h~6 h	5	1306	0	0.878	0.327
	4 h~6 h versus ≥8 h	4	432	0	0.914	0.497
	12 h versus 24 h	2	235	0	/	/
Hematoma	/	10	2256	0	0.415	0.788
	2 h versus 4 h~6 h	2	325	0	/	/
	3 h~4 h versus 5 h~6 h	4	1359	0	0.899	1
	4 h~6 h versus ≥8 h	3	482	0	0.781	0.602
Back pain	/	6	569	72.063	0.621	0.851
	3 h~4 h versus 5 h~6 h	2	111	69.263	/	/
	4 h~6 h versus ≥8 h	2	223	0	/	/
	12 h versus 24 h	2	235	61.813	/	/
Pseudoaneurysm	/	3	1277	0	0.941	0.602
Urinary retention	/	4	719	73.87	0.476	0.497
	12 h versus 24 h	2	235	0	/	/
Pain at the puncture site	/	2	812	21.31	/	/

## 4. Discussion

To the best of our knowledge, this study is the first systematic review involving both English and Chinese studies, using the information from 14 randomized controlled trials with 2653 participants, to assess the effect of early mobilization on patients’ complications after trans-femoral cardiac catheterization. Nowadays, early mobilization is strongly recommended in to shorten the length of hospital stay and enhance recovery after surgery (ERAS) [50]. It has been associated with a reduced risk of insulin resistance, gastrointestinal complications, thromboembolism, and de-conditioning of the cardiovascular, respiratory, and musculoskeletal systems, especially for elderly patients. However, the lack of a standard term for “early mobilization” may lead to delayed mobilization [51, 52].

Our meta-analysis demonstrated that early mobilization of patients after cardiac catheterization via the femoral artery is practicable, and was associated with a lower incidence of back pain. That was in line with the two similar reviews by Mohammady *et al*. [24, 53]. Prolonged supine bed rest causes pressure to be exerted continuously onto the same back muscle, which inevitably results in back pain [54]. However, early mobilization did not necessarily reduce the risk of urinary retention, pain at the puncture site, and vascular complications such as bleeding, hematoma, and pseudoaneurysm, which were relatively consistent with previous reviews [24, 53, 55, 56]. It was confirmed that VCD were an effective hemostatic measure to prevent bleeding, surpassing manual compression and sandbags [57, 58, 59]. Even though the usage of VCD was ruled out from the study selection process, we still found early mobilization did not increase the risk of vascular complications at the puncture site. As opposed to our finding, a recent network meta-analysis conducted by Busca *et al*. [30] indicated a lower risk of hematoma at a shorter bed rest duration and a higher risk at a longer duration. That was surprising and not representative, as the effect on potential confounding variables of VCD may explain these findings. Traditional pressure dressing and manual compression, which can stretch across or circumferentially envelope the torso to constitute an absorbent layer over the sterile dressing sites by the elastic adhesive bandage, can press the dead space to reduce the risk of hematoma and seroma formation [60, 61]. In contrast, the pressure of VCD is hard to measure and even causes damage to the puncture site which can accelerate the formation of a hematoma.

In this meta-analysis, we classified the bed rest duration into four categories (2 h versus 4 h~6 h, 3 h~4 h versus 5 h~6 h, 4 h~6 h versus ≥8 h, 12 h versus 24 h) in which the short bed rest duration was regarded as early mobilization in each category based on the comprehensive literature review, in summary, the early mobilization times varied from 2 h to 6 h except two studies happening in mainland China set as 12 h. The longer early mobilization time may result from the prudent notion of traditional Chinese culture and fewer attempts related to ERAS for Chinese cardiac catheterization patients. Our subgroup analysis results resembling previous reviews showed early mobilization significantly released back pain in the 3 h~4 h versus 5 h~6 h and 12 h versus 24 h categories and urinary retention in the 12 h versus 24 h category [24, 53]. We found patient bed rests for 12 h related to a lower risk of urinary retention than bed rest for 24 h. That may be because longer resting time in bed results in lower neuronal output activities from the same sacral roots as the bladder and lower limbs [62].

Cardiac catheterization, routinely using heparin and aspirin directed by the managing clinician, has the risk of vascular complications. As is reported, lower heparin doses, such as 25 UI/kg/h, have an apparent half-life of 30 minutes, whereas higher doses of 100 and 400 UI/kg/h are associated with half-lives of 60 minutes and 150 minutes, respectively [63]. Combining the results of this meta-analysis, we suggested patients who underwent trans-femoral cardiac catheterization could mobilize after 2 h~4 h bed rest for the sake of safety and comfort.

In a word, we perceived all the included 14 studies as a moderate to low heterogeneity because the $I^{2}$ index depicted in Table 2 were lower than 75%, which suggested our findings could be regarded as robust. But some statistically significant results from subgroup analysis regarding back pain and urinary retention outcomes should be assumed to be overweighted because of the limited numbers and data from the same research team. In addition, two eligible Chinese mainland randomized controlled trials enriched the sources of evidence compared to previous studies, resulting in not only the credibility of the conclusion being highlighted but the applicability of the findings being more extensive.

## 5. Limitations

Our study has some limitations. Firstly, the definition of bleeding, hematoma formation, pseudoaneurysm, and urinary retention varied among studies, and pain is a self-perceived experience. It may lower our confidence in the results with the increasing risk of heterogeneity. Secondly, the small number of studies gave us less access to fully evaluate the effect of early mobilization on patients’ complication outcomes, especially specifying the reliable effect on some outcomes based on different bed rest duration is impossible. Finally, we could not conduct a more comprehensive subgroup analysis with incomplete information on heparin usage and sheath sizes from original studies. We could only infer that all the included studies adopted hemostasis protocols, whether they are well-standardized protocols is not able to confirm. They are also sources of heterogeneity. Further well-designed study is needed, and the findings of our meta-analysis should be interpreted with caution.

## 6. Conclusions

In conclusion, the findings from our meta-analysis approved the current proposition that shortening bed rest duration is beneficial to patients who have undergone cardiac catheterization trans-femoral artery; it is possible to underscore the safety and effectiveness of early mobilization after 2 h~4 h bed rest without more risk of vascular complications and discomfort. Even if the benefits of early mobilization are considered to be beyond the perceived risks, significant barriers, including fears of vascular complications and the complexity of the specific surgical site, can interfere with its proceeding [64]. According to the reality and circumstances of different surgical sites, the perspectives and initiatives on positively helping patients move early based on the convincing evidence are the beginning of the continued success of ERAS.

## Data Availability

All data points generated or analyzed during this study are included in this article and there are no further underlying data necessary to reproduce the results.

## Supplementary Material

Supplementary material associated with this article can be found, in the online version, at https://doi.org/10.31083/j.rcm2505152.
