# Supplementary material for: Early Mobilization after Cardiac Catheterization via Femoral Artery: A Systematic Review and Meta-Analysis
Source: Rev Cardiovasc Med. 2024 Apr 30;25(5):152. doi: 10.31083/j.rcm2505152 (PMC11267210; doi:10.31083/j.rcm2505152)
Supplement: Supplementary file 1 [file 2153-8174-25-5-152-s1.zip › 2153-8174-25-5-152-s1/Supplementary Material/Supplementary material.docx]

Supplementary Material 1.

Search strategies

1.Pubmed

(((((((((((((((((((((“Cardiac Catheters”[Mesh]) OR Cardiac Catheter*[Text Word]) OR Catheter*, Cardiac [Text Word]) OR Intracardiac Catheter*[Text Word]) OR Catheter*, Intracardiac [Text Word]) OR Heart Catheter*[Text Word]) OR Catheter*, Heart [Text Word])) OR (((“Angiography”[Mesh]) OR Angiograph*[Text Word]) OR Arteriograph*[Text Word])) OR ((((((((“Percutaneous Coronary Intervention”[Mesh]) OR Percutaneous Coronary Intervention*[Text Word]) OR Coronary Intervention*, Percutaneous [Text Word]) OR Intervention*, Percutaneous Coronary [Text Word]) OR Percutaneous Coronary Revascularization*[Text Word]) OR Coronary Revascularization*, Percutaneous [Text Word]) OR Revascularization*, Percutaneous Coronary [Text Word]))) OR (((“Coronary Angiography”[Mesh]) OR Coronary Angiograph*[Text Word]) OR Angiograph*, coronary [Text Word])) OR (((((((((“Angioplasty”[Mesh]) OR Angioplast*[Text Word]) OR Endoluminal Repair*[Text Word]) OR Repair*, Endoluminal [Text Word]) OR Percutaneous Transluminal Angioplast*[Text Word]) OR Angioplast*, Percutaneous Transluminal [Text Word]) OR Transluminal Angioplast*, Percutaneous [Text Word]) OR Angioplast*, Transluminal [Text Word]) OR Transluminal Angioplast*[Text Word])) OR (((((“Femoral Artery”[Mesh]) OR Femoral Artery [Text Word]) OR Femoral Arteries [Text Word]) OR Artery, Femoral [Text Word]) OR Arteries, Femoral [Text Word])) OR ((“Punctures”[Mesh]) OR Puncture*[Text Word])) OR ((((((((((“Cardiac Catheterization”[Mesh]) OR Cardiac Catheterization*[Text Word]) OR Catheterization*, Cardiac [Text Word]) OR Cardiac Catheterisation*[Text Word]) OR Catheterisation*, Cardiac [Text Word]) OR Heart Catheterization*[Text Word]) OR Heart Catheterisation*[Text Word]) OR Catheterization, Heart [Text Word]) OR Catheterisation, Heart [Text Word]))) OR transfemoral approach*[Text Word]) OR ((((((“Angioplasty, Balloon”[Mesh]) OR Angioplast*, Balloon [Text Word]) OR Balloon Angioplast*[Text Word]) OR Transluminal Arterial Dilation*[Text Word]) OR Arterial Dilation*, Transluminal [Text Word]))) OR ((((((((((“Angioplasty, Balloon, Coronary”[Mesh]) OR Angioplast*, Balloon, Coronary [Text Word]) OR Transluminal Coronary Balloon Dilation [Text Word]) OR Coronary Balloon Angioplast*[Text Word]) OR Balloon Angioplast*, Coronary [Text Word]) OR Angioplast*, Coronary Balloon [Text Word]) OR Coronary Angioplast*, Transluminal Balloon [Text Word]) OR Balloon Dilation, Coronary Artery [Text Word]) OR Angioplast*, Transluminal, Percutaneous Coronary [Text Word]) OR Percutaneous Transluminal Coronary Angioplast*[Text Word])).

And

((((((((((((((((((((“Early Ambulation”[Mesh]) OR Early Ambulation [Text Word]) OR Ambulation, Early [Text Word]) OR Accelerated Ambulation [Text Word]) OR Ambulation, Accelerated [Text Word]) OR Early Mobilization [Text Word]) OR Mobilization, Early [Text Word]) OR Ambulation [Text Word]) OR Early [Text Word]) OR Moblization [Text Word]) OR Mobilisation [Text Word]) OR Immediate Mobilization [Text Word]) OR Immediate Mobilisation [Text Word]) OR Early Mobilisation [Text Word]) OR Mobilisation, Early [Text Word]))) OR ((“Posture”[Mesh]) OR Posture*[Text Word])) OR (((“Walking”[Mesh]) OR walking [Text Word]) OR ambulation [Text Word])))).

And

((((((((((“Bed Rest”[Mesh]) OR bed rest [Text Word]) OR bed rests [Text Word]) OR Rest, Bed [Text Word]) OR Bedrest*[Text Word]) OR Rests, Bed [Text Word]))) OR (((“Immobilization”[Mesh]) OR Immobilization [Text Word]) OR Immoblisation [Text Word])) OR ((((“Rest”[Mesh]) OR rest [Text Word]) OR rests [Text Word]) OR resting time [Text Word]))).

And

((((((((((((((((((((((((((((((((“Back Pain”[Mesh]) OR back pain [Text Word]) OR Back Pains [Text Word]) OR Pain, Back [Text Word]) OR Pains, Back [Text Word]) OR Backache [Text Word]) OR Backaches [Text Word]) OR Back Ache [Text Word]) OR Ache, Back [Text Word]) OR Aches, Back [Text Word]) OR Back Aches [Text Word])) OR (((“Hematoma”[Mesh]) OR Hematoma*[Text Word]) OR Haematoma*[Text Word])) OR ((((((“Hemorrhage”[Mesh]) OR Hemorrhag*[Text Word]) OR Haemorrhage*[Text Word]) OR Active bleeding*[Text Word]) OR Bleeding*[Text Word]))) OR ((((((“Patient Satisfaction”[Mesh]) OR Satisfaction, Patient [Text Word]) OR Satisfaction, Patients [Text Word]) OR Patient Satisfaction [Text Word]) OR Patients Satisfaction [Text Word]))) OR ((((“Urination Disorders”[Mesh]) OR Urination Disorder*[Text Word]) OR Disorder*, Urination [Text Word]) OR Micturition disorder*[Text Word]) OR (((((((((“Postoperative Care”[Mesh]) OR Postoperative Care [Text Word]) OR Care, Postoperative [Text Word]) OR Postoperative Procedure*[Text Word]) OR Procedure*, Postoperative [Text Word]) OR post-operative care [Text Word]) OR Care, Post-operative [Text Word]) OR post-operative procedure*[Text Word]) OR Procedure*, Post-operative [Text Word])) OR ((((((“Postoperative Complications”[Mesh]) OR Complication*, Postoperative [Text Word]) OR Postoperative Complication*[Text Word]) OR Complication*, Post-operative [Text Word]) OR Post-operative Complication*[Text Word]))) OR ((((((((((“Postoperative Period”[Mesh]) OR Postoperative Period [Text Word]) OR Postoperative Periods [Text Word]) OR Post-operative Period [Text Word]) OR Post-operative Periods [Text Word]) OR Period, Postoperative [Text Word]) OR Periods, Postoperative [Text Word]) OR Period, Post-operative [Text Word]) OR Periods, Post-operative [Text Word]))) OR ((“Patient Satisfaction”[Mesh]) OR Patient* Satisfaction [Text Word])) OR (((((“Consumer Satisfaction”[Mesh]) OR Consumer Satisfaction [Text Word]) OR Satisfaction, Consumer [Text Word]) OR Consumer Preference*[Text Word]) OR Preference*, Consumer [Text Word])) OR (((“Hospitalization”[Mesh]) OR Hospitalization*[Text Word]) OR Hospitalisation*[Text Word])) OR ((“Safety”[Mesh]) OR safety [Text Word])) OR (((“Patient Safety”[Mesh]) OR Patient Safet*[Text Word]) OR Safet*, Patient [Text Word])) OR (((“Vascular Diseases”[Mesh]) OR vascular disease*[Text Word]) OR Disease*, Vascular [Text Word])) OR ((((((“Peripheral Vascular Diseases”[Mesh]) OR Peripheral Vascular Disease*[Text Word]) OR Disease*, Peripheral Vascular [Text Word]) OR Vascular Disease*, Peripheral [Text Word]) OR Peripheral Angiopath*[Text Word]) OR Angiopath*, Peripheral [Text Word])) OR (((“Aneurysm”[Mesh]) OR Aneurysm*[Text Word]))) OR ((((((“Aneurysm, False”[Mesh]) OR Aneurysm*, False [Text Word]) OR False Aneurysm*[Text Word]) OR Pseudoaneurysm*[Text Word]) OR Pseudo-aneurysm*[Text Word]))) OR (((((“Arteriovenous Fistula”[Mesh]) OR Arteriovenous Fistula*[Text Word]) OR Fistula*, Arteriovenous [Text Word]) OR Arteriovenous Aneurysm*[Text Word]) OR Aneurysm*, Arteriovenous [Text Word])) OR ((“complications” [Subheading]) OR complication*[Text Word])) OR (((((“Length of Stay”[Mesh]) OR Length of Stay [Text Word]) OR Stay Length*[Text Word]) OR Hospital Stay*[Text Word]) OR Stay*, Hospital [Text Word])) OR effect*[Text Word]).

2. EMBASE

‘cardiac catheter’/exp OR ‘cardiac catheter’ OR ‘heart catheter’/exp OR ‘angiography’/exp OR ‘angiography’ OR ‘percutaneous coronary intervention’/exp OR ‘percutaneous coronary intervention’ OR ‘percutaneous transluminal angioplasty’/exp OR ‘percutaneous transluminal angioplasty’ OR ‘angiocardiography’/exp OR ‘angiocardiography’ OR ‘angioplasty’/exp OR ‘angioplasty’ OR ‘transluminal angiocardiography’ OR ‘femoral angiography’/exp OR ‘femoral angiography’ OR ‘puncture’/exp OR ‘puncture’ OR ‘heart catheterization’/exp OR ‘heart catheterization’ OR ‘cardiac catheterization’/exp OR ‘cardiac catheterization’ OR (cardiac AND (‘catheterisation’/exp OR catheterisation)) OR ‘transfemoral approach’ OR (‘angioplasty’/exp OR angioplasty AND (‘balloon’/exp OR balloon)) OR (transluminal AND coronary AND (‘balloon’/exp OR balloon) AND (‘dilatation’/exp OR dilatation)).

And

(early AND (‘ambulation’/exp OR ambulation) OR ‘mobilization’/exp OR mobilization OR ‘ambulation’/exp OR ‘ambulation’ OR (early AND (‘mobilization’/exp OR mobilization)) OR ‘posture’/exp OR ‘posture’ OR (body AND (‘posture’/exp OR posture)) OR ‘walking’/exp OR walking) AND.

(‘bed rest’/exp OR ‘bed rest’ OR ‘rest’/exp OR ‘rest‘OR ‘immobilization’/exp OR immobilization OR ‘immobilisation’/exp OR immobilisation).

And

(‘back’/exp OR back AND (‘pain’/exp OR pain) OR ‘backache’/exp OR backache OR (low AND (‘back’/exp OR back) AND (‘pain’/exp OR pain)) OR ‘hematoma’/exp OR hematoma OR ‘haematoma’/exp OR haematoma OR ‘hemorrhage’/exp OR hemorrhage OR ‘haemorrhage’/exp OR haemorrhage OR ‘bleeding’/exp OR bleeding OR ‘patient satisfaction’/exp OR ‘patient satisfaction’ OR (‘urination’/exp OR urination AND (‘disorder’/exp OR disorder)) OR ‘micturition disorder’/exp OR ‘micturition disorder’ OR (postoperative AND care) OR ‘complication’/exp OR complication OR ‘consumer’/exp OR consumer OR ‘hospitalization’/exp OR hospitalization OR hospitalisation OR ‘patient safety’/exp OR ‘patient safety’ OR ‘consumer satisfaction’/exp OR ‘consumer satisfaction’ OR (vascular AND (‘disease’/exp OR disease)) OR (peripheral AND vascular AND (‘disease’/exp OR disease)) OR ‘aneurysm’/exp OR aneurysm OR ‘false aneurysm’/exp OR ‘false aneurysm’ OR ‘pseudoaneurysm’/exp OR pseudoaneurysm OR ‘arteriovenous fistula’/exp OR ‘arteriovenous fistula’ OR (lenght AND of AND stay) OR (‘hospital’/exp OR hospital AND stay) OR effect) AND [embase]/lim.

3. Cochrane Database of Systematic Reviews

| ID | Search |
| --- | --- |
| #1 | MeSH descriptor: [Cardiac Catheterization] explode all trees |
| #2 | MeSH descriptor: [Cardiac Catheters] explode all trees |
| #3 | MeSH descriptor: [Angiography] explode all trees |
| #4 | MeSH descriptor: [Percutaneous Coronary Intervention] explode all trees |
| #5 | MeSH descriptor: [Punctures] explode all trees |
| #6 | #1 or #2 or #3 or #4 or #5 |
| #7 | MeSH descriptor: [Early Ambulation] explode all trees |
| #8 | MeSH descriptor: [Walking] explode all trees |
| #9 | MeSH descriptor: [Posture] explode all trees |
| #10 | #7 or #8 or #9 |
| #11 | MeSH descriptor: [Bed Rest] explode all trees |
| #12 | MeSH descriptor: [Rest] explode all trees |
| #13 | #11 or #12 |
| #14 | MeSH descriptor: [Back Pain] explode all trees |
| #15 | MeSH descriptor: [Hematoma] explode all trees |
| #16 | MeSH descriptor: [Hemorrhage] explode all trees |
| #17 | MeSH descriptor: [Patient Satisfaction] explode all trees |
| #18 | MeSH descriptor: [Urination Disorders] explode all trees |
| #19 | MeSH descriptor: [Postoperative Complications] explode all trees |
| #20 | MeSH descriptor: [Hospitalization] explode all trees |
| #21 | MeSH descriptor: [Vascular Diseases] explode all trees |
| #22 | MeSH descriptor: [Aneurysm] explode all trees |
| #23 | MeSH descriptor: [Aneurysm, False] explode all trees |
| #24 | MeSH descriptor: [Arteriovenous Fistula] explode all trees |
| #25 | #14 or #15 or #16 or #17 or #18 or #19 or #20 or #21 or #22 or #23 or #24 |
| #26 | #6 and #10 and #13 and #25 |

4.CINAHL

| S55 | S14 AND S21 AND S26 AND S54 |
| --- | --- |
| S54 | S27 OR S28 OR S29 OR S30 OR S31 OR S32 OR S33 OR S34 OR S35 OR S36 OR S37 OR S38 OR S39 OR S40 OR S41 OR S42 OR S43 OR S44 OR S45 OR S46 OR S47 OR S48 OR S49 OR S50 OR S51 OR S52 OR S53 |
| S53 | “effect*” |
| S52 | (MH “Length of Stay”) OR (MH “Bed Occupancy”) OR “length of stay” |
| S51 | “complication” |
| S50 | (MH “Arteriovenous Fistula”) OR “arteriovenous fistula” |
| S49 | “pseudoaneurysm” OR (MH “Aneurysm, False”) |
| S48 | (MH “Aneurysm+”) OR “aneurysm” |
| S47 | (MH “Peripheral Vascular Diseases+”) OR “peripheral Vascular Disease” |
| S46 | (MH “Vascular Diseases+“) OR “vascular disease” |
| S45 | (MH “Patient Safety+”) OR “patient safety” |
| S44 | (MH “Safety+”) OR “safety” |
| S43 | (MH “Hospitalization+”) OR “hospitalization” |
| S42 | (MH “Catheter-Related Complications”) OR “Catheter-Related Complication” |
| S41 | (MH “Postoperative Complications”) OR “postoperative complication” |
| S40 | (MH “Postoperative Period”) OR “postoperative period” |
| S39 | (MH “Postoperative Care+”) OR “postoperative care” OR (MH “Postoperative Hemorrhage”) |
| S38 | “micturition” |
| S37 | (MH “Urination Disorders”) OR “urination disorders” |
| S36 | (MH “Consumer Satisfaction”) OR “consumer satisfaction” |
| S35 | (MH “Patient Satisfaction”) OR “patient satisfaction” |
| S34 | “active bleeding” |
| S33 | “bleeding” OR (MH “Bleeding Time”) |
| S32 | (MH “Hemorrhage+”) OR “hemorrhage” |
| S31 | “hematoma” |
| S30 | (MH “Hematoma+”) OR “haematoma” |
| S29 | “backache” |
| S28 | (MH “Low Back Pain”) OR “low back pain” |
| S27 | (MH “Back Pain+”) OR “back pain” |
| S26 | S22 OR S23 OR S24 OR S25 |
| S25 | “resting time” |
| S24 | (MH “Immobilization”) OR “immobilization” |
| S23 | “rest” |
| S22 | (MH “Bed Rest”) OR “bed rest” |
| S21 | S15 OR S16 OR S17 OR S18 OR S19 OR S20 |
| S20 | (MH “Walking+”) OR “walking” |
| S19 | (MH “Posture+”) OR “posture” |
| S18 | “mobilization” |
| S17 | “ambulation” |
| S16 | “early mobilization” |
| S15 | (MH “Early Ambulation”) OR “early ambulation” |
| S14 | S1 OR S2 OR S3 OR S4 OR S5 OR S6 OR S7 OR S8 OR S9 OR S10 OR S11 OR S12 OR S13 |
| S13 | (MH “Angioplasty, Transluminal, Percutaneous Coronary”) OR (MH “Angioplasty, Balloon+”) OR “percutaneous transluminal coronary angioplast*” |
| S12 | “transluminal arterial dilatation” OR (MH “Arterial Puncture”) |
| S11 | (MH “Angioplasty, Balloon+”) OR “angioplasty balloon” |
| S10 | “transfemoral approach” |
| S9 | (MH “Punctures+”) OR “puncture” |
| S8 | (MH “Femoral Artery”) OR “femoral artery” |
| S7 | “coronary angiograph*” OR (MH “Coronary Angiography”) OR (MH “Angioplasty+”) OR “angioplast*” OR (MH “Angioplasty, Balloon+”) |
| S6 | “coronary angiograph*” OR (MH “Coronary Angiography”) |
| S5 | “Percutaneous Coronary revascularization” |
| S4 | (MH “Angioplasty, Transluminal, Percutaneous Coronary”) OR “percutaneous coronary intervention” |
| S3 | (MH “Angiography+”) OR “angiograph*” |
| S2 | (MH “Catheterization, Central Venous+”) OR “heart catheter*” |
| S1 | (MH “Heart Catheterization+”) OR “cardiac catheter*” |

5. SCOPUS

(ALL (cardiac catheter*) OR ALL(heart catheter*) OR ALL(angiograph*) OR ALL(percutaneous coronary intervention*) OR ALL(percutaneous transluminal angioplast*) OR ALL(angiocardiograph*) OR ALL(angioplast*) OR ALL(transluminal angiocardiograph*) OR ALL(femoral angiograph*) OR ALL(puncture*) OR ALL(transfemoral approach*) OR ALL(angioplasty balloon*) OR ALL(transluminal coronary balloon dilatation*)) AND (ALL(early ambulation) OR ALL(early mobilization) OR ALL(early mobilisation) OR ALL(ambulation) OR ALL(mobilization) OR ALL(mobilisation) OR ALL(posture*) OR ALL(body posture*) OR ALL(walking)) AND (ALL(bed rest*) OR ALL(rest*) OR ALL(immobilisation*) OR ALL(immobilization*)) AND (ALL(back pain) OR ALL(backache) OR ALL(low back pain) OR ALL(hematoma*) OR ALL(haematoma*) OR ALL(hemorrhage*) OR ALL(haemorrhage*) OR ALL(bleeding*) OR ALL(patient* satisfaction*) OR ALL(urination disorder*) OR ALL(micturition disorder*) OR ALL(postopeartive care) OR ALL(post-operative care) OR ALL(complication*) OR (consumer*) OR ALL(hospitalization) OR ALL(hospitalisation) OR ALL(patient* safety) OR ALL(consumer* satisfaction) OR ALL(vascular disease*) OR ALL(peripheral vascular disease*) OR ALL(aneurysm*) OR ALL(false aneurysm*) OR ALL(pseudoaneurysm*) OR ALL(arteriovenous fistula*) OR ALL(lenght of stay) OR ALL(hospital stay) OR ALL(effect*)) AND (LIMIT-TO(SUBJAREA, “MEDI”) OR LIMIT-TO(SUBJAREA, “HEAL”) OR LIMIT-TO(SUBJAREA, “NURS”)) AND (EXCLUDE(SUBJAREA, “AGRI”) OR EXCLUDE(SUBJAREA, “ARTS”) OR EXCLUDE(SUBJAREA, “BIOC”) OR EXCLUDE(SUBJAREA, “BUSI”) OR EXCLUDE(SUBJAREA, “CENG”) OR EXCLUDE(SUBJAREA, “CHEM”) OR EXCLUDE(SUBJAREA, “COMP”) OR EXCLUDE(SUBJAREA, “DENT”) OR EXCLUDE(SUBJAREA, “ECON”) OR EXCLUDE(SUBJAREA, “ENER”).

1. Web of Science

| #1 | TS= ("Cardiac Catheter*" OR "Catheter*, Cardiac" OR "Intracardiac Catheter*") OR TS= ("Catheter*, Intracardiac" OR "Heart Catheter*" OR "Catheter*, Heart") |
| --- | --- |
| #2 | TS= ("Angiograph*" OR "Angiogram*" OR "Arteriograph*" OR "Angiocardiography") |
| #3 | TS= ("Percutaneous Coronary Intervention*" OR "Coronary Intervention*, Percutaneous") OR TS= ("Intervention*, Percutaneous Coronary" OR "Percutaneous Coronary Revascularization*") OR TS= ("Coronary Revascularization*, Percutaneous" OR "Revascularization*, Percutaneous Coronary") OR TS= ("Atherectomy, Coronary") |
| #4 | TS= ("Angioplast*" OR "Endoluminal Repair*" OR "Repair*, Endoluminal" OR "Percutaneous Transluminal Angioplasty") OR TS= ("Angioplasty, Percutaneous Transluminal" OR "Transluminal Angioplasty, Percutaneous") OR TS= ("Angioplasty, Transluminal" OR "Transluminal Angioplasty") |
| #5 | TS= ("Angioplasty, Balloon, Laser-Assisted" OR "Atherectomy, Coronary" OR "Atherectomy") |
| #6 | TS=  ("Coronary Angiograph*" OR "Angiograph*, Coronary") |
| #7 | TS= ("Femoral Arter*" OR "Arter*, Femoral") |
| #8 | TS= ("Puncture*" OR "Micropuncture*" OR "Paracentesis") |
| #9 | TS= ("Cardiac Catheterization*" OR "Catheterization*, Cardiac" OR "Catheterization*, Heart") OR TS= ("Heart Catheterization*" OR "Catheterization, Swan-Ganz") |
| #10 | TS= ("Angioplasty, Balloon" OR "Dilation*, Transluminal Arterial" OR "Arterial Dilation*, Transluminal") OR TS= ("Transluminal Arterial Dilation*" OR "Balloon Angioplasty") |
| #11 | TS= ("Angioplasty, Balloon, Coronary" OR "Transluminal Coronary Balloon Dilation") OR TS= ("Balloon Dilation, Coronary Artery" OR "Angioplast*, Coronary Balloon") OR TS= ("Balloon Angioplast*, Coronary" OR "Coronary Balloon Angioplast*") OR TS= ("Coronary Angioplasty, Transluminal Balloon" OR "Angioplasty, Transluminal, Percutaneous Coronary") OR TS= ("Percutaneous Transluminal Coronary Angioplasty" OR "Angioplasty, Transluminal") |
| #12 | #11 OR #10 OR #9 OR #8 OR #7 OR #6 OR #5 OR #4 OR #3 OR #2 OR #1 |
| #13 | TS= ("Early Ambulation" OR "Accelerated Ambulation" OR "Ambulation, Accelerated") |
| #14 | TS= ("Posture*" OR "Supine Position") |
| #15 | TS= ("Walking" OR "Ambulation" OR "Exercise") |
| #16 | #15 OR #14 OR #13 |
| #17 | TS= ("Bed Rest*" OR "Rest*, Bed") |
| #18 | TS= ("Immobilization" OR "Hypokinesia, Experimental" OR "Experimental Hypokinesia*") OR TS= ("Hypokinesias, Experimental" OR "Restraint, Physical") |
| #19 | TS= ("rest*") |
| #20 | #19 OR #18 OR #17 |
| #21 | TS= ("Back Pain*" OR "Pain*, Back" OR "Backache*" OR "Back Ache*" OR "Ache*, Back") OR TS= ("Back Pain without Radiation" OR "Vertebrogenic Pain Syndrome") OR TS= ("Pain Syndrome*, Vertebrogenic" OR "Syndrome*, Vertebrogenic Pain") |
| #22 | TS= ("Hematoma*") |
| #23 | TS= ("Hemorrhage*" OR "Bleeding") |
| #24 | TS= ("Patient Satisfaction" OR "Satisfaction, Patient") |
| #25 | TS= ("Urination Disorder*" OR "Disorder*, Urination") |
| #26 | TS= ("Postoperative Care" OR "Care, Postoperative" OR "Postoperative Procedure*") OR TS= ("Procedure*, Postoperative") |
| #27 | TS= ("Postoperative Complication*" OR "Complication*, Postoperative") |
| #28 | TS= ("Postoperative Period*" OR "Period*, Postoperative") |
| #29 | TS= ("consumer satisfaction" OR "Satisfaction, Consumer") |
| #30 | TS= ("Hospitalization*" OR "Hospitalisation*") |
| #31 | TS= ("Safet*") |
| #32 | TS= ("Patient Safet*" OR "Safet*, Patient") |
| #33 | TS= ("Peripheral Vascular Disease*" OR "Disease, Peripheral Vascular" OR "Vascular Disease, Peripheral") OR TS= ("Peripheral Angiopath*" OR "Angiopath*, Peripheral" OR "Peripheral Angiopathy") |
| #34 | TS= ("Aneurysm*" OR "Fusiform Aneurysm*" OR "Aneurysm*, Fusiform" OR "Saccular Aneurysm") |
| #35 | TS= ("Aneurysm*, False" OR "False Aneurysm*" OR "Pseudoaneurysm*") |
| #36 | TS= ("Arteriovenous Fistula*" OR "Fistula*, Arteriovenous" OR "Arteriovenous Aneurysm") OR TS= ("Aneurysm, Arteriovenous") |
| #37 | TS= ("Length of Stay" OR "Stay Length*" OR "Hospital Stay*" OR "Stay*, Hospital") |
| #38 | #38 OR #37 OR #36 OR #35 OR #34 OR #33 OR #32 OR #31 OR #30 OR #29 OR #28 OR #27 OR #26 OR #25 OR #24 OR #23 OR #22 OR #21 |
| #39 | #39 AND #20 AND #16 AND #12 |

1. **Wan Fang Database**

(("Cardiac Catheter" or "Catheter, Cardiac" or "Catheter, Heart" or "Intracardiac Catheter" or "Catheter, Swan-Ganz" or "Punctures" or "Angioplasty, Balloon, Coronary" or "Angioplasty, Balloon" or "Percutaneous Transluminal Angioplast" or "Angiography" or "Angiography, Coronary" or "Intervention") and "Femoral Artery") and ("Daily activity" or "Ambulation" or "Immobilization" or "position" or "timing") and ("Back Pain" or "Hematoma" or "Hemorrhage" or "Urination Disorders" or "uroschesis" or "Postoperative Care" or "Perioperative Care" or "Aneurysm, False" or "False Aneurysm" or "Hospital Stay" or "Arteriovenous Fistula" or "Arteriovenous Aneurysm" or "Patient safe" or "Patient Satisfaction") (In Chinese)

1. **Chinese Science and Technology Periodical Database (VIP)**

(M=Cardiac Catheter + M=Catheter, Heart + M=Catheter, Cardiac + M=Intracardiac Catheter + M=Catheter, Swan-Ganz + M=Punctures + M=Angioplasty, Balloon + M=Angioplasty, Transluminal, Percutaneous Coronary + M=Angioplasty, Transluminal, Balloon + M=Transluminal Angioplast*, Percutaneous + M=Coronary Angiography + M=Angiography, Coronary + M=Intervention) * M=Femoral Artery * M=(timing + Hemorrhage +Back Pain +Hematoma + Urination Disorders + uroschesis + Perioperative Care + Postoperative Care + Aneurysm, False + False Aneurysm + Hospital Stay + Arteriovenous Fistula + Arteriovenous Aneurysm + Patient safe + Patient Satisfaction + Nursing) * M=(Immobilization + position) (In Chinese)

1. **China National Knowledge Infrastructure (CNKI)**

SU=('Cardiac Catheter'+'Catheter, Cardiac'+'Catheter, Heart'+'Intracardiac Catheter'+'Catheter, Swan-Ganz'+'Punctures'+'Angioplasty, Balloon'+'Angioplasty, Transluminal, Balloon'+'Transluminal Angioplasty, Percutaneous'+'Coronary Angiography'+'Angiography, Coronary'+'Intervention') *'Femoral Artery'*('Timing'+'Hemorrhage'+'Back Pain'+'Hematoma'+'Urination Disorders'+'uroschesis'+'Postoperative Care'+'Aneurysm, False'+'False Aneurysm'+'Hospital Stay'+'Arteriovenous Fistula'+'Arteriovenous Aneurysm'+'Patient Satisfaction'+'Patient safe'+'Nursing')*('Immobilization'+'position') (In Chinese)

1. **JBI**

(cardiac catheterization.mp. [mp=text, heading word, subject area node, title] ) OR (percutaneous coronary intervention.mp. [mp=text, heading word, subject area node, title])
